# Development and Preliminary Validation of the Breath Motor Pattern Index (BMPI): An Observational Measure of Respiratory Pattern Quality in Children

**DOI:** 10.3390/children13060759

**Published:** 2026-05-29

**Authors:** Aleksandra Moluszys, Łukasz Mański, Mirella Kozakiewicz, Marek Niedoszytko, Eliza Wasilewska

**Affiliations:** 1 Gdansk Medical Academy of Applied Sciences, 80-335 Gdansk, Poland; 2 Gdansk University of Physical Education and Sport, 80-336 Gdansk, Poland; mirela.kozakiewicz@awf.gda.pl; 3 Department of Allergology, Medical University of Gdansk, 80-210 Gdansk, Poland; marek.niedoszytko@gumed.edu.pl

**Keywords:** respiratory motor pattern, breathing assessment, pediatric physiotherapy, postural control, respiratory motor organization, psychometric validation

## Abstract

**Highlights:**

**What are the main findings?**

- Breathing is an organized motor activity integrated with postural and motor control systems, rather than an isolated physiological function.
- Evidence from neurophysiological, developmental, and pediatric studies shows that breathing is coordinated, task-dependent, and functionally linked to movement.
- Current clinical assessments fail to capture breathing pattern organization, supporting the need for a functional, pattern-based tool such as the Breath Motor Pattern Index (BMPI).

**What are the implications of the main findings?**

- Breathing should be assessed as a functional motor pattern integrated with postural control, rather than as an isolated physiological parameter.
- Current clinical evaluation in children may overlook important aspects of breathing organization, potentially limiting the effectiveness of physiotherapy and rehabilitation strategies.
- The findings support the development and implementation of pattern-based assessment tools, such as the Breath Motor Pattern Index (BMPI), to improve diagnosis and guide targeted interventions.

**Abstract:**

**Background/Objectives**: Breathing is increasingly recognized as an integral component of the motor system, interacting with postural control and movement. Despite this, clinical assessment of respiratory function in children remains largely limited to physiological parameters, with relatively few tools available to evaluate breathing as an organized motor pattern. The aim of this study was to develop and preliminarily validate the Breath Motor Pattern Index (BMPI), an observational tool designed to assess the organization of respiratory motor patterns in children. **Methods**: A scoping review was conducted to identify key components of respiratory motor pattern organization. Based on these findings, the BMPI was developed and evaluated in a cohort of 210 children aged 0–72 months, divided into three groups: healthy controls, children with neurological conditions, and children with respiratory disorders. Inter-rater and test–retest reliability was assessed using intraclass correlation coefficients (ICC). Measurement error was quantified using the standard error of measurement (SEM) and minimal detectable change (MDC95). Construct-related validity was examined through correlations with the Gross Motor Function Measure (GMFM-88) and comparisons between clinical groups. **Results**: The BMPI showed high inter-rater reliability (ICC = 0.998) and test–retest reliability (ICC = 0.999), with low measurement error (SEM = 0.55; MDC95 = 1.53). A weak but statistically significant correlation with GMFM-88 was observed (rho = 0.23, *p* < 0.001). BMPI scores differed significantly between groups (*p* < 0.001), with lower values observed in the neurological group and higher values in the pulmonary group. **Conclusions**: The BMPI appears to be a promising observational tool with potential clinical applicability for assessing respiratory motor pattern organization in children. The findings support the conceptualization of breathing as an integrated component of the motor system while highlighting the need for further psychometric and longitudinal validation studies. Future research should further investigate the responsiveness of the BMPI as well as its potential utility in clinical decision-making and therapeutic monitoring.

## 1. Introduction

Contemporary approaches to rehabilitation, particularly in pulmonology and pediatrics, predominantly interpret breathing in terms of improving ventilatory function. In many therapeutic protocols, breathing is treated as a tool to enhance tissue oxygenation, airway clearance, or respiratory parameters, while it is less frequently viewed as an integrated motor behavior closely linked to postural and movement organization [1]. Although clinically justified, this perspective may overlook the broader role of breathing as an organizing element of the motor system.

Despite the growing body of research on breathing assessment, most available methods rely on the analysis of physiological parameters using specialized measurement equipment [2]. Tools such as spirometry are often not accessible in routine physiotherapy practice, and their application in pediatric populations is further limited by the child’s age and the inability to obtain reliable cooperation. Consequently, functional assessment of breathing during therapy and monitoring of therapeutic outcomes remain significantly constrained [3].

Several observational tools for breathing assessment have previously been described, including instruments focused on breathing pattern analysis or respiratory dysfunction screening, such as the Breathing Pattern Assessment Tool (BPAT). However, most existing tools are primarily designed for adult populations or focus predominantly on respiratory symptoms and ventilatory mechanics rather than the integration of breathing with postural control and motor organization. In contrast, the BMPI was developed to assess breathing as a multidimensional motor pattern within a developmental and functional pediatric context.

From a functional perspective, breathing fulfills the criteria of a motor pattern—it represents an organized, sequential neuromuscular activity with multisegmental and multiplanar characteristics, modulated according to task demands [4]. It is an integral component of motor behavior and remains closely linked to postural control [5]. From a developmental standpoint, respiratory movements are among the earliest emerging motor patterns in ontogenesis, and their organization provides the foundation for the development of postural and motor functions [6]. Disruptions in this primary pattern may hinder the acquisition of motor milestones and lead to the persistence of inefficient movement strategies [7].

In clinical practice, breathing disturbances are still predominantly interpreted as secondary to neurological pathology. However, an increasing number of patients present with postural and movement impairments without a clear neurological origin, while their medical history often reveals recurrent or prolonged respiratory conditions [8]. This suggests that breathing is insufficiently recognized as a primary factor in shaping motor patterns. A disrupted breathing pattern may influence muscle tone regulation, stabilization strategies, and postural control, ultimately affecting overall movement quality [9].

In response to this clinical gap and the need for a more functional approach to breathing assessment, the Breath Motor Pattern Index (BMPI) was developed.

The aim of this study was to synthesize the available tools for breathing assessment and to propose a novel instrument enabling the systematic evaluation of breathing as a motor pattern and its integration with postural function. The study was designed as a scale validation process, focused on assessing its psychometric properties, with the goal of supporting clinical assessment and therapy planning.

## 2. Materials and Methods

### 2.1. Study Procedures

The study was designed as a multi-stage methodological project aimed at the development and evaluation of the psychometric properties of the Breath Motor Pattern Index (BMPI). The investigation was prospective in nature and consisted of three stages.

The first stage involved a scoping review conducted in accordance with PRISMA-ScR guidelines. Its aim was to map the current state of knowledge on breathing as a motor pattern in children, identify gaps in existing assessment tools, and define the key components of respiratory motor pattern organization.

The second stage consisted of the development of the BMPI based on the findings of the literature review and theoretical assumptions regarding breathing as a motor pattern. This stage included the definition of assessment domains, development of the scale structure and scoring criteria, as well as evaluation of content validity by an expert panel and pilot testing of the tool.

The third stage involved the psychometric validation of the BMPI in clinical settings. The study had an observational, cross-sectional design and included standardized assessment procedures. Each participant underwent a single assessment session to evaluate inter-rater reliability, and a subset of participants was reassessed to determine test–retest reliability.

No randomization procedures were implemented, as the study was not designed to evaluate the effects of an intervention but to assess the measurement properties of the developed tool. Although formal study-wide blinding procedures were not implemented, raters were blinded to each other’s scores and were not informed about participant group allocation during scoring.

The sample size was determined based on feasibility and participant availability during the study period. The study population included children with diverse clinical profiles to allow evaluation of the BMPI across a broad functional spectrum.

Given the methodological aim of the study, no formal a priori hypotheses were formulated and no statistical power calculation was performed. The study was not intended for hypothesis testing but for the assessment of reliability, measurement error, and construct validity of the BMPI.

The study design was aligned with established methodological recommendations for the development and validation of measurement instruments, including the COSMIN guidelines.

### 2.2. Stage 1: Scoping Review

A scoping review was conducted in accordance with the PRISMA-ScR guidelines to systematically map current evidence on breathing as a motor pattern in children and its relationship with motor development and postural control [10]. The primary aim of this stage was to identify conceptual gaps in existing assessment approaches and define key components of respiratory motor pattern organization to inform the development of the BMPI.

The literature search was performed in PubMed, Scopus, Google Scholar, and Web of Science. The search strategy included combinations of keywords and MeSH terms related to breathing and motor function, such as “breathing”, “respiratory pattern”, “motor pattern”, “postural control”, and “motor development”, combined using Boolean operators (AND, OR). The search strategy was adapted to the indexing structure of each database. Detailed search strategies for each database, including full search strings and search dates, are provided in Supplementary Materials Table S2.

Publications from January 2000 to December 2025 were considered. The review included studies focusing on pediatric populations (0–18 years) and addressing respiratory pattern development, breathing from a motor perspective, and the integration of breathing with posture and movement. Eligible publications included observational studies, review articles, and conceptual papers available in full-text, peer-reviewed form with DOI or PMID identifiers. Articles published in English and Polish were included.

Exclusion criteria comprised studies focusing exclusively on adult populations, publications addressing respiratory diseases without reference to motor function, animal studies, and articles not related to postural control, movement, or child development.

All identified records were imported into Zotero (version 8.0.5) reference management software, where duplicate records were removed prior to screening. Titles and abstracts were screened to identify potentially relevant studies, followed by full-text assessment based on predefined eligibility criteria. Reference lists of included articles were additionally screened to identify further relevant publications.

For each included study, data were extracted regarding study design, participant characteristics, and key aspects of breathing assessment in relation to motor function.

As the scoping review was conducted as a preliminary conceptual stage intended to support instrument development rather than evidence synthesis for clinical recommendations, no protocol registration was performed. Consistent with PRISMA-ScR methodology, the review aimed to identify conceptual and clinically relevant components of respiratory motor organization rather than to evaluate intervention effectiveness or establish levels of evidence.

The scoping review and conceptual development phase were initiated prior to participant recruitment and ethics approval, as these stages did not involve human participant data collection.

**Figure 1:**
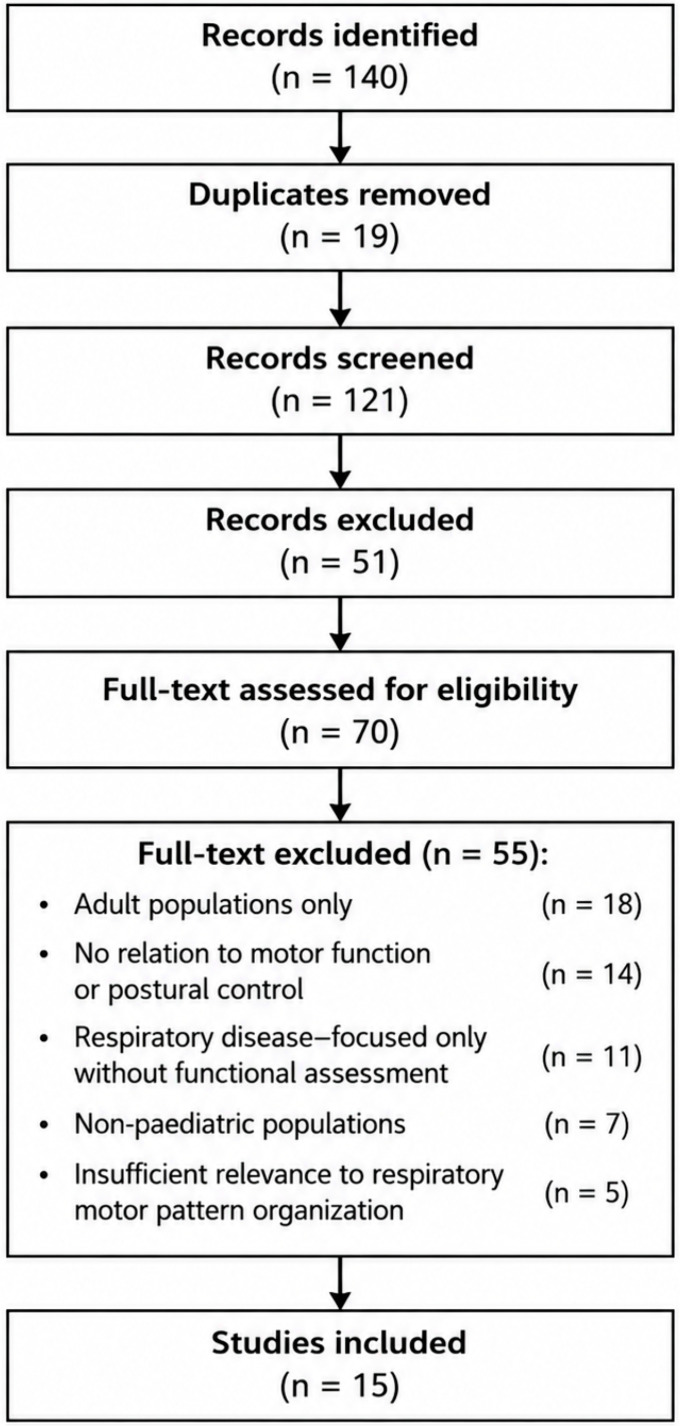
Flow diagram of the study selection process for the scoping review. A total of 140 records were identified through database searching (PubMed, Scopus, Web of Science) and additional sources (Google Scholar). After removal of 19 duplicate records, 121 articles were screened based on titles and abstracts, of which 51 were excluded. Seventy full-text articles were assessed for eligibility, and 15 studies were included in the final conceptual synthesis.

The database search yielded 140 records. After removal of 19 duplicate records, 121 articles were screened based on titles and abstracts, of which 51 were excluded. Seventy articles were assessed for full-text eligibility, and 15 publications met the inclusion criteria and were included in the conceptual analysis (Figure 1).

Data from the included studies were extracted and summarized in a structured format, including study design, population characteristics, assessment methods, and key findings. The detailed characteristics of all included studies are presented in Table 1.

Study selection was performed by two reviewers, with disagreements resolved through discussion to reach consensus.

Due to the exploratory nature of the scoping review, no formal quality assessment or quantitative synthesis was performed. Instead, findings were synthesized using a descriptive and conceptual approach to identify key components of respiratory motor pattern organization and highlight gaps in current clinical assessment approaches. Given the nature of this approach, the synthesis is inherently interpretive and should be considered exploratory.

Following the synthesis of the available literature, the identified characteristics of breathing in relation to motor development and postural control were analysed to define key components of respiratory motor pattern organization. Based on this analysis, a conceptual framework of breathing as a motor pattern was established. These components were subsequently operationalized into the domains of the Breath Motor Pattern Index (BMPI).

Given the limited and heterogeneous nature of the available evidence, this framework should be considered exploratory and hypothesis-generating. It is intended to provide a structured basis for the systematic assessment of breathing as a motor pattern and to support the development of clinically applicable evaluation tools in paediatric physiotherapy.

### 2.3. Stage 2: Development of the BMPI

The development of the Breath Motor Pattern Index (BMPI) assumed that breathing can be conceptualized as a motor pattern when it meets specific organizational criteria. A motor pattern was defined as being characterized by: (1) organized and sequential neuromuscular activity, (2) multisegmental and multiplanar kinematics, (3) context-dependent modulation, and (4) interaction with other motor tasks [6]. These assumptions constituted the conceptual foundation for the construction of the tool. Accordingly, the latent construct underlying the BMPI was defined as the qualitative organization of breathing as an integrated motor behavior associated with postural control, neuromuscular coordination, adaptive modulation, and compensatory motor strategies. A detailed theoretical and neurophysiological framework linking BMPI domains with their corresponding constructs and clinical observations is presented in Supplementary Materials Table S1.

The first step involved the identification of key characteristics of breathing which, based on the literature review and theoretical framework, could be interpreted as components of respiratory motor pattern organization. Attention was given to aspects of breathing that are clinically observable, functionally relevant, and related to postural control and movement organization.

In the next stage, the identified components were organized into four assessment domains. The first domain addressed initiation and rhythmicity of breathing, reflecting the level of organization and continuity of neuromuscular activity. The second domain focused on thoraco-abdominal coordination as a clinical expression of multisegmental movement organization. The third domain concerned the ability to adapt the breathing pattern to changing postural and task-related demands, corresponding to context-dependent modulation. The fourth domain addressed the integration of breathing with postural control and the presence of compensatory strategies, capturing the interaction between breathing and other motor functions.

The preliminary BMPI consisted of 16 observational items distributed equally across four domains (four items per domain), with each item scored on a three-point ordinal scale (0–2 points), resulting in a total possible score ranging from 0 to 32.

The four-domain structure of the BMPI was conceptually derived from the scoping review findings and expert consensus during the development phase. Although the instrument consists of 16 items organized into four theoretically defined domains, no exploratory or confirmatory factor analysis was performed at this stage. Therefore, the proposed domain structure should currently be interpreted as conceptual rather than empirically validated.

**Figure 2:**
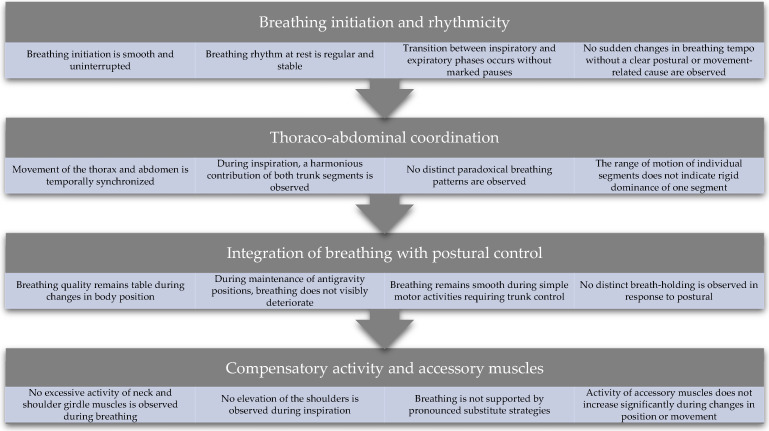
The conceptual structure of the BMPI. The complete BMPI scoring framework is provided in Supplementary Materials Table S3.

Subsequently, specific observational items were developed for each domain. The construction of items was aimed at capturing the quality of breathing organization rather than the intensity or magnitude of respiratory movement. Each item was designed to reflect a clearly defined, clinically observable feature of the breathing pattern that could be assessed without the use of specialized equipment. At this stage, emphasis was placed on functional relevance, clarity, and applicability in routine paediatric clinical practice. The conceptual structure of the BMPI, including its domains and scoring framework, is presented in Figure 2.

The preliminary structure of the BMPI, including the assessment domains, observational items, scoring system, and assessment procedure, was reviewed by an expert panel consisting of four specialists: two physiotherapists with expertise in pediatric and respiratory physiotherapy, one physician specializing in allergology, and one physician specializing in internal medicine and allergology. The experts evaluated the clinical relevance, clarity, and applicability of the proposed domains and items. Based on their feedback, modifications were introduced to improve the clarity, organization, and clinical usability of the scale.

Prior to the main study, pilot testing of the BMPI was conducted in a small group of children representing the target age range. The pilot phase involved trained pediatric physiotherapists experienced in respiratory and neurodevelopmental assessment. The aim of the pilot testing was to evaluate the clarity of item descriptions, feasibility of observation under routine clinical conditions, scoring consistency, and overall usability of the instrument. Based on pilot observations and expert feedback, minor wording adjustments and clarifications to several scoring descriptors were introduced to improve interpretability and clinical applicability.

A scoring system was then developed using a three-point ordinal scale (0–2 points), reflecting the level of motor pattern organization. A score of 0 indicated absence or clear disorganization of the feature, a score of 1 indicated partial presence with variability or context dependence, and a score of 2 indicated a well-organized, stable, and functionally integrated pattern. This approach was adopted to enable structured qualitative assessment in clinical settings.

In parallel, the principles of the assessment procedure were established. The BMPI was designed as an observational tool based on the child’s spontaneous breathing in different positions and functional contexts with varying postural demands. The assessment was intended to be conducted under natural conditions, without providing breathing-related instructions, in order to avoid influencing the spontaneous respiratory pattern. The selection of observational positions was intended to enable evaluation of both the stability of the breathing pattern and its adaptability to changing postural contexts.

As a result, a preliminary version of the BMPI was developed as a tool for the systematic clinical assessment of breathing as a motor pattern. The structure of the scale was designed to enable both overall evaluation of respiratory motor pattern organization and qualitative analysis across its individual dimensions.

### 2.4. BMPI Assessment Procedure

BMPI assessment is observational and based on the evaluation of the child’s spontaneous breathing pattern. The procedure does not require specialized equipment and is designed to be applicable in standard clinical settings.

All observations are conducted in a controlled and calm environment to ensure the child’s comfort and to minimize external influences on breathing behaviours. The child is preferably assessed without clothing that may interfere with visual inspection of thoraco-abdominal movements. No instructions regarding breathing are provided during the assessment to avoid voluntary modulation of the respiratory pattern. Breathing is observed across positions with varying postural demands, including supine, sitting, and—when developmentally appropriate—standing or simple functional activity. Each position is observed for approximately 30–60 s.

Visual assessment is the primary method of evaluation. When necessary, gentle palpation of the chest and abdominal regions may be applied to support observation, if it does not alter the child’s natural behaviours.

Scoring is based on the predominant breathing pattern observed within each position. In cases of variability across positions, the lowest consistently observed level of motor pattern organization is recorded to reflect the most functionally relevant limitation. Higher BMPI scores indicate more efficient and integrated respiratory motor organization, whereas lower scores reflect disorganized patterns and the presence of compensatory strategies.

### 2.5. Stage 3: Psychometric Validation of the BMPI

#### 2.5.1. Participants

The study included a total of 210 children aged 0–72 months. Participants were recruited from a pediatric physiotherapy outpatient setting and were assigned to three groups based on clinical characteristics: (1) typically developing children (n = 70), (2) children with neurological conditions affecting motor development and postural control, including cerebral palsy, genetic syndromes, hypotonia, and global developmental delay (n = 70), and (3) children with chronic or recurrent respiratory disorders, including disorders of mucociliary clearance, cystic fibrosis, bronchopulmonary dysplasia, recurrent obstructive bronchitis, and other inherited pulmonary diseases (n = 70).

To account for developmental variability in respiratory motor pattern organization, participants were further stratified into five age groups: 0–11 months, 12–23 months, 24–35 months, 36–47 months, and 48–72 months. This stratification was based on key stages of ontogenetic development of respiratory function and its integration with postural control and motor function. In early infancy, breathing is closely linked to the maturation of basic postural mechanisms, whereas in later stages it becomes progressively integrated with trunk stabilization, functional movement, and context-dependent modulation. The applied age grouping enabled capturing stage-specific changes in respiratory motor pattern organization.

Inclusion criteria were defined as age within the specified range (0–72 months), clinically stable condition at the time of assessment, absence of acute respiratory exacerbation or hospitalization, and the ability to undergo observational evaluation of breathing in at least one body position. Exclusion criteria included acute medical conditions affecting respiratory function, lack of cooperation preventing assessment, and conditions significantly limiting postural evaluation beyond the scope of the study.

Participants were recruited using a convenience sampling strategy during the study period. The sample size was determined based on feasibility and participant availability, in accordance with methodological recommendations for preliminary validation studies.

All assessments were conducted in a child-friendly environment designed to minimize stress and ensure natural behaviours during observation. Attention was given to maintaining a calm atmosphere and allowing the child to remain in a comfortable state, as emotional distress may influence spontaneous breathing patterns and compromise the validity of the assessment.

The inclusion of children with diverse clinical profiles ensured sufficient variability in respiratory motor pattern organization, which is essential for the evaluation of construct validity and other measurement properties of the BMPI.

Informed consent was obtained from all participants’ legal guardians. The study protocol was approved by the relevant institutional ethics committee (No. KB/151/2026).

#### 2.5.2. Study Design

The study was designed as a multi-stage, observational validation study aimed at evaluating the psychometric properties of the Breath Motor Pattern Index (BMPI). The investigation was prospective in nature and included standardized assessment procedures conducted in clinical settings.

The validation phase had a cross-sectional design and involved the assessment of participants using the BMPI under controlled conditions. Each participant underwent a single assessment session for the evaluation of inter-rater reliability. Assessments were performed independently by three pairs of trained paediatric physiotherapists, each pair evaluating the same participants in parallel while remaining blinded to each other’s ratings. Additionally, a subset of participants (n = 10) was independently assessed by all three pairs of evaluators, allowing both pairwise and multi-rater agreement to be evaluated.

To determine test–retest reliability, repeated assessments were conducted in a subset of participants following a defined time interval, during which no therapeutic intervention affecting respiratory function was introduced. The same standardized BMPI assessment protocol was applied at both time points.

All assessments were conducted by physiotherapists experienced in paediatric respiratory and neurodevelopmental evaluation. To ensure consistency, all evaluators followed a predefined and standardized assessment procedure.

No randomization or blinding of participants was implemented, as the study was not designed to evaluate the effects of an intervention but to assess the measurement properties of a newly developed clinical instrument.

The study included children with diverse clinical profiles to ensure variability in respiratory motor pattern organization, which is essential for the evaluation of construct validity. Inclusion of participants across different developmental stages further enabled assessment of the scale across a broad functional spectrum.

The study was conducted in accordance with methodological recommendations for validation studies of clinical measurement instruments, including the COSMIN guidelines.

#### 2.5.3. Statistical Analysis

Statistical analysis was performed using Jamovi software (version 2.7.19.0). The level of statistical significance was set at *p* < 0.05.

The distribution of variables was assessed using the Shapiro–Wilk test and visual inspection of histograms. Normally distributed variables were presented as mean ± standard deviation (SD), while non-normally distributed variables were described using median and interquartile range (IQR). Due to the non-normal distribution of the data, non-parametric statistical tests were applied.

Inter-rater and test–retest reliability of the BMPI were evaluated using the intraclass correlation coefficient (ICC) with 95% confidence intervals. A two-way random-effects model with absolute agreement and single measures was applied, as raters were considered representative of a larger population and exact agreement between measurements was required. ICC values were interpreted as follows: <0.5 poor, 0.5–0.75 moderate, 0.75–0.9 good, and >0.9 excellent reliability.

Measurement error was quantified using the standard error of measurement (SEM) and the minimal detectable change at the 95% confidence level (MDC95). SEM was calculated as: SEM = SD × √(1 − ICC) and MDC95 was calculated as: MDC95 = 1.96 × √2 × SEM.

Item-level agreement was additionally evaluated using weighted Cohen’s kappa.

Construct validity was assessed by examining the relationship between BMPI and GMFM-88 scores using Spearman’s rank correlation coefficient.

Group differences in BMPI scores were analysed using the Kruskal–Wallis test. Post hoc pairwise comparisons were performed using Dunn’s test with Bonferroni correction. Effect size for group differences was calculated using epsilon-squared (ε^2^) to estimate the magnitude of the observed effect.

Floor and ceiling effects were evaluated and considered present if more than 15% of participants achieved the lowest or highest possible score. No missing data were identified in the dataset, and all participants were included in the final analysis.

## 3. Results

### 3.1. Sample Characteristics

**Figure 3:**
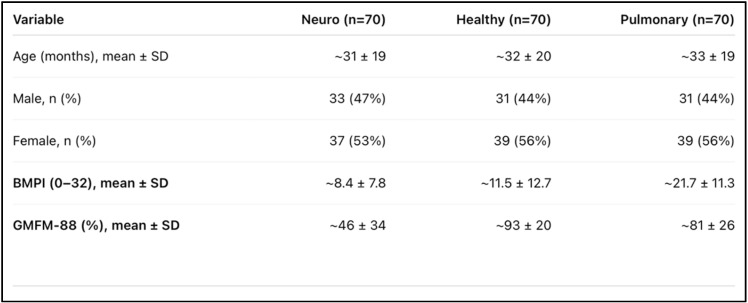
Demographic and clinical characteristics of the study population and distribution of BMPI scores across clinical groups.

Participant characteristics are presented in Figure 3. A total of 210 children aged 0–72 months were included and evenly distributed across three clinical groups (n = 70 per group).

The mean age was comparable across groups, with 31 ± 19 months in the neurological group, 32 ± 20 months in the control group, and 33 ± 19 months in the pulmonary group. No significant differences in age or sex distribution were observed between groups (*p* > 0.05). The proportion of males was 47% in the neurological group and 44% in both the control and pulmonary groups.

Descriptive analysis of BMPI scores revealed clear differences between groups. The neurological group demonstrated the lowest mean BMPI score (8.4 ± 7.8), followed by the pulmonary group (11.5 ± 12.7), while the control group exhibited the highest scores (21.7 ± 11.3).

A similar pattern was observed for GMFM-88 scores. The neurological group showed the lowest functional performance (46 ± 34%), whereas higher values were observed in the pulmonary group (81 ± 26%) and the control group (93 ± 20%).

Detailed demographic and clinical characteristics, including the distribution of BMPI and GMFM-88 scores, are summarized in Figure 3.

**Inter-Rater Reliability**

Inter-rater reliability of the BMPI suggested a high level of agreement across evaluators. Results are summarized in Table 2. Assessments were conducted live by three pairs of trained pediatric physiotherapists with experience in respiratory and neurodevelopmental evaluation. Raters observed the assessments simultaneously but scored independently and were blinded to each other’s ratings and group allocation. Discussion between raters during scoring was not permitted. Participants included in the reliability analyses were selected based on the availability of complete paired and repeated assessments conducted under standardized observational conditions. Test–retest reliability was assessed by repeating the evaluation by the same rater under comparable observational conditions.

For the pairwise analysis, baseline assessments from children evaluated independently by two raters were included (n = 90, after exclusion of one case due to allocation inconsistency). The intraclass correlation coefficient (ICC) for the total BMPI score was 0.998 (95% CI: 0.996–0.999), suggesting a very high level of agreement between raters.

At the domain level, inter-rater reliability also appeared to be high. ICC values were 0.991 (95% CI: 0.983–0.996) for domain B, 0.993 (95% CI: 0.985–0.999) for domain T, 0.999 (95% CI: 0.997–1.000) for domain P, and 0.997 (95% CI: 0.993–0.999) for domain C.

Additionally, a subset of participants (n = 10) was independently assessed by all three rater pairs, enabling multi-rater reliability analysis. In this overlapping subset, the ICC for the total BMPI score was 0.999 (95% CI: 0.995–1.000), which further suggests consistency of BMPI scoring across multiple evaluators. Domain-specific ICC values in the multi-rater subset were 0.987 (95% CI: 0.935–1.000) for domain B, 0.997 (95% CI: 0.981–1.000) for domain T, 1.000 (95% CI: 1.000–1.000) for domain P, and 1.000 (95% CI: 1.000–1.000) for domain C.

Item-level agreement, assessed using weighted Cohen’s kappa, ranged from 0.961 to 1.000, indicating substantial to almost perfect agreement across BMPI items.

**Figure 4:**
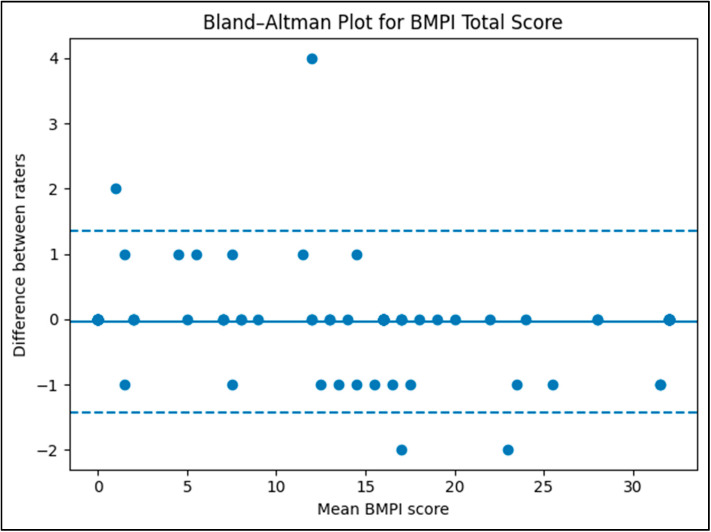
Bland–Altman plot illustrating agreement between raters for the BMPI total score. The solid line represents the mean difference (bias), while the dashed lines indicate the 95% limits of agreement. The distribution of differences suggests no systematic bias and a narrow range of variability between raters.

Agreement between raters was further examined using Bland–Altman analysis (Figure 4). The mean bias was −0.04, suggesting minimal systematic differences between raters, while the 95% limits of agreement ranged from −1.45 to 1.36, indicating a relatively narrow range of variability.

Overall, these findings suggest that the BMPI may provide consistent and reproducible measurements of respiratory motor pattern organization, with limited variability attributable to rater-dependent factors.

### 3.2. Test–Retest Reliability

Test–retest reliability of the BMPI was assessed to evaluate the temporal stability of the measurement over a 7-day interval, as summarized in Table 3. Repeated assessments were performed in a subset of participants (n = 90), using the same standardized BMPI assessment protocol. No therapeutic intervention affecting respiratory function was introduced between assessments.

The intraclass correlation coefficient (ICC) for the total BMPI score was 0.999 (95% CI: 0.998–1.000), suggesting a very high level of agreement between the two assessment time points.

At the domain level, test–retest reliability also appeared to be high. ICC values were 1.000 (95% CI: 0.999–1.000) for domain B, 0.999 (95% CI: 0.997–1.000) for domain T, 1.000 (95% CI: 1.000–1.000) for domain P, and 1.000 (95% CI: 1.000–1.000) for domain C.

Overall, these findings suggest that BMPI scores remained highly stable across repeated assessments, with limited variability attributable to short-term measurement fluctuation.

### 3.3. Measurement Error

Measurement error of the BMPI was assessed using the standard error of measurement (SEM) and the minimal detectable change at the 95% confidence level (MDC95), based on inter-rater reliability estimates.

The SEM for the total BMPI score was 0.55, indicating a low level of measurement variability. The MDC95 was calculated as 1.53, suggesting that a change greater than this value may reflect a real change in respiratory motor pattern organization rather than measurement error.

These findings suggest that the BMPI demonstrates a relatively small measurement error. However, its sensitivity to clinically meaningful changes over time requires further longitudinal validation.

### 3.4. Construct Validity

Construct validity of the BMPI was examined by assessing its relationship with motor function and its ability to differentiate between clinical groups.

A statistically significant positive correlation was observed between BMPI and GMFM-88 scores (rho = 0.23, *p* < 0.001), suggesting a weak association between respiratory motor pattern organization and gross motor function.

**Figure 5:**
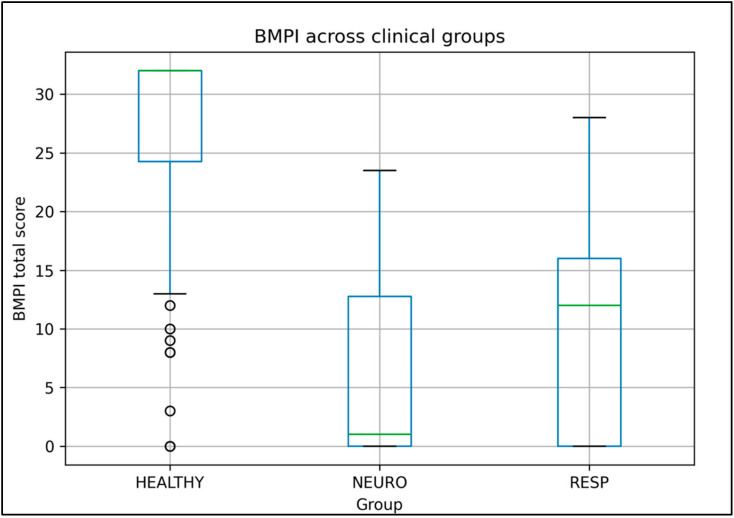
Distribution of BMPI total scores across clinical groups. The boxplots illustrate median values, interquartile ranges, and variability within each group. Differences between groups suggest variability in respiratory motor pattern organization across clinical populations.

Differences in BMPI scores between clinical groups were also identified. The Kruskal–Wallis test revealed statistically significant differences across groups (H = 102.70, *p* < 0.001, ε^2^ = 0.49), indicating a large effect size. Post hoc analysis indicated that children in the neurological group demonstrated lower BMPI scores compared to both the control and pulmonary groups, while the control group exhibited the highest scores. Differences in BMPI scores between clinical groups are illustrated in Figure 5, which visually supports the observed group differences.

Overall, these findings suggest that the BMPI may capture aspects of respiratory motor organization that are partially related to motor function and differ across clinical populations.

### 3.5. Floor and Ceiling Effects

Floor and ceiling effects were observed, with 26.7% of participants achieving the lowest possible BMPI score and 21.4% achieving the highest possible score. These values exceed the commonly accepted threshold of 15%, suggesting clustering of scores at both extremes of the scale. This pattern may reflect the inclusion of clinically distinct groups with markedly different levels of motor and respiratory function.

## 4. Discussion

### 4.1. Principal Findings

The present study aimed to develop and preliminarily validate the Breath Motor Pattern Index (BMPI) as an observational tool for assessing respiratory motor pattern organization in children. The findings of this study suggest that the BMPI demonstrates a high level of inter-rater reliability and temporal stability, indicating that the assessment may be reproducible across evaluators and over short time intervals.

The very high inter-rater and test–retest reliability estimates observed in this study should be interpreted with caution. Such values may reflect the structured and operationally simplified nature of the BMPI, standardized assessment conditions, and the training of raters. In addition, the BMPI was intentionally designed as a clinically observable tool to improve scoring consistency across evaluators. However, the obtained reliability estimates may also have been influenced by limited variability within the sample or clustering of observations. In addition, the presence of floor and ceiling effects may have contributed to reduced score variability, which could partially inflate reliability estimates. Future multicenter studies involving broader clinical heterogeneity and more variable clinical environments are therefore necessary to further verify the robustness of these psychometric properties. Further validation in independent and heterogeneous populations is also warranted.

In addition, the relatively low measurement error suggests that the relatively low measurement error suggests potential utility for detecting changes beyond measurement variability; however, responsiveness to developmental and therapeutic change has not yet been established.

Construct validity analyses indicated that BMPI scores were weakly but significantly associated with gross motor function, as measured by the GMFM-88. This finding suggests that respiratory motor pattern organization may be partially related to overall motor performance, while still representing a distinct aspect of functional organization. This observation is consistent with previous work emphasizing the interaction between breathing and postural control within the motor system.

Furthermore, BMPI scores appeared to differentiate between clinical groups, with lower scores were observed in children with neurological conditions, intermediate scores in the pulmonary group, and the highest scores in typically developing controls. This pattern may reflect differences in the organization and adaptability of the respiratory motor pattern across populations with varying functional characteristics.

Overall, these findings suggest that the BMPI may provide a clinically relevant measure of respiratory motor pattern organization; however, further research is needed to confirm its broader applicability and sensitivity.

### 4.2. BMPI as a Motor Pattern Construct

The conceptual foundation of the BMPI assumes that breathing can be understood as a motor pattern rather than solely a physiological process. In this perspective, respiratory activity is organized through coordinated neuromuscular interactions, integrated with postural control and other motor functions.

In the present study, this assumption was operationalized into four domains reflecting key characteristics of motor patterns: initiation and rhythmicity, thoraco-abdominal coordination, adaptability to postural and task demands, and integration with postural control and compensatory strategies. These domains were designed to capture both the organization and flexibility of the respiratory motor pattern, rather than isolated aspects of ventilation.

This approach is consistent with theoretical and experimental work suggesting that breathing is functionally linked with trunk stabilization and postural regulation. The diaphragm has been described as playing a dual role in both ventilation and postural control, contributing to the regulation of intra-abdominal pressure and trunk stability [20]. From this perspective, respiratory function cannot be fully understood without considering its interaction with the motor system.

The inclusion of multiple domains in the BMPI reflects the complexity of respiratory motor organization. Rather than assuming a single “optimal” breathing pattern, the scale was designed to account for context-dependent variability, which is particularly relevant in paediatric populations, where motor and respiratory functions are still developing.

At the same time, it should be acknowledged that the conceptualization of breathing as a motor pattern remains an evolving framework. The BMPI represents an attempt to translate this theoretical perspective into a clinically applicable observational tool; however, further research is needed to refine the construct and to better understand its physiological and biomechanical correlates.

### 4.3. Relationship with Motor Function

In the present study, a statistically significant but weak association was observed between BMPI scores and gross motor function as assessed by the GMFM-88. This finding should be interpreted with caution.

At first glance, the relatively low correlation coefficient may suggest a limited relationship between respiratory motor pattern organization and overall motor performance. However, this result may also reflect the fact that the BMPI and GMFM-88 assess related but fundamentally distinct constructs. While the GMFM-88 is designed to evaluate the execution of gross motor tasks, the BMPI focuses on the qualitative organization of the respiratory motor pattern, including its integration with postural control and adaptability.

From this perspective, the weak correlation observed in the present study may be considered consistent with the conceptual framework of the BMPI. Rather than representing a general measure of motor performance, the BMPI may capture a more specific dimension of motor–respiratory organization that is not directly reflected in global functional motor scales. Therefore, the observed correlation should not be interpreted as evidence of substantial construct overlap, but rather as preliminary support for a partial relationship between respiratory motor organization and gross motor function.

This interpretation is supported by previous research indicating that respiratory function contributes to postural control and movement organization through anticipatory and stabilizing mechanisms of the diaphragm [12]. Additionally, the relationship between breathing and motor function may not be strictly linear. Respiratory motor organization may influence movement quality and postural control in a subtle and context-dependent manner that is not fully captured by global motor assessments such as the GMFM-88.

Taken together, these findings suggest that the BMPI and GMFM-88 provide complementary rather than overlapping information, supporting the potential value of including respiratory motor pattern assessment as part of a broader functional evaluation in children.

### 4.4. Discriminative Ability Across Clinical Groups

In the present study, BMPI scores appeared to differentiate between clinical groups, with statistically significant differences observed across populations with distinct functional profiles.

Children in the neurological group demonstrated lower BMPI scores compared to both the control and pulmonary groups, suggesting a reduced level of respiratory motor pattern organization. This finding may reflect the impact of impaired postural control, altered muscle tone, and disrupted motor coordination commonly observed in neurological conditions, which may influence the integration of breathing with motor function. This interpretation is consistent with previous research indicating that children with neurological disorders often present with altered respiratory patterns, reduced respiratory muscle function, and impaired coordination between breathing and movement [21,22].

In contrast, children in the pulmonary group exhibited higher BMPI scores, in some cases exceeding those observed in the control group. This observation may suggest that, despite the presence of respiratory pathology, these children may develop compensatory strategies that support the organization and efficiency of the respiratory motor pattern. Alternatively, it may reflect differences in the underlying mechanisms affecting respiratory function, which are not primarily related to motor control.

Importantly, BMPI scores should not be interpreted as direct indicators of respiratory disease severity or general health status. The BMPI was designed to assess the organization and adaptability of the respiratory motor pattern rather than pulmonary efficiency or symptom burden. Therefore, differences observed between groups may reflect distinct patterns of motor-respiratory organization rather than a simple healthy-versus-pathological distinction. In addition, the relatively broad developmental age range included in the control group may have contributed to substantial variability in BMPI scores, particularly given the ongoing maturation of postural and respiratory integration during early childhood.

The ability of the BMPI to distinguish between these groups may support its potential utility as a clinical tool for identifying differences in respiratory motor organization across paediatric populations. At the same time, it should be acknowledged that the observed differences may be influenced by the heterogeneity of the study groups and the complex interaction between respiratory and motor systems.

Overall, these findings suggest that BMPI may provide clinically relevant information regarding the organization of breathing in children with different functional characteristics, although further research is needed to better understand the mechanisms underlying these differences.

### 4.5. Developmental Perspective

The interpretation of BMPI results should be considered within the context of developmental changes in respiratory and motor function. In early childhood, breathing is not yet fully stabilized and undergoes dynamic changes as part of the ontogenetic maturation of the motor system.

From this perspective, variability in respiratory motor patterns may represent a physiological feature rather than a dysfunction, particularly in younger children. The development of breathing is closely linked to the maturation of postural control, trunk stability, and neuromuscular coordination, which evolve progressively during the first years of life [23].

Importantly, the respiratory system in children differs structurally and functionally from that of adults. The paediatric chest wall is more compliant, the rib cage is positioned more horizontally, and respiratory muscles are still developing, which together influence breathing mechanics and efficiency. Lung development continues postnatally, with ongoing alveolarization and maturation of pulmonary structures throughout early childhood [24].

In addition, the function of the diaphragm evolves with age, transitioning from a primarily respiratory role in infancy toward a more integrated role in both ventilation and postural control as the motor system matures [25]. This developmental progression may contribute to changes in the organization and adaptability of the respiratory motor pattern over time.

This developmental context is particularly relevant when interpreting BMPI scores across age groups. The absence of a single “optimal” breathing pattern in children suggests that respiratory motor organization should be understood as a flexible and adaptive process rather than a fixed configuration. Consequently, lower or more variable BMPI scores in younger children may not necessarily indicate pathology but may instead reflect an earlier stage of functional development.

At the same time, the interaction between respiratory and motor systems may become more organized and efficient with age, as postural control mechanisms mature and the integration of breathing with movement becomes more refined. This may partially explain the variability observed within and between clinical groups in the present study.

These considerations highlight the importance of interpreting BMPI results within a developmental framework. The BMPI may therefore have potential utility not only as a diagnostic tool but also for longitudinal observation of respiratory motor organization over time. However, these potential applications require further longitudinal validation studies.

### 4.6. Methodological Considerations and Critical Appraisal

Several methodological aspects of the present study should be considered when interpreting the findings.

First, floor and ceiling effects were observed, with a relatively high proportion of participants achieving the lowest and highest possible BMPI scores. This may suggest clustering at the extremes of the scale, which could limit the sensitivity and discriminative ability of the instrument in detecting subtle differences within highly homogeneous subgroups. At the same time, this pattern may reflect the inclusion of clinically distinct populations characterized by markedly different levels of motor and respiratory function. These findings may also indicate the need for future refinement of selected items or age-specific adaptations to improve sensitivity across different developmental and clinical profiles.

Second, the assessment of construct validity was based on the relationship between BMPI and GMFM-88 scores. While GMFM-88 is a widely used and validated measure of gross motor function, it does not directly assess respiratory or postural integration mechanisms [26]. Therefore, the observed weak correlation should be interpreted with caution, as it may reflect differences in the constructs measured rather than a limitation of the BMPI itself.

Third, no gold standard measure for respiratory motor pattern organization currently exists, which limits the possibility of evaluating criterion validity. As a result, the validation process relied on indirect approaches, including construct validity and known-groups comparisons. While this is consistent with current recommendations for the development of novel clinical instruments, it may limit the extent to which the BMPI can be directly compared with existing measures. Moreover, although the conceptual framework of the BMPI assumes integration between respiratory organization and postural control, no direct objective assessment of postural function was included in the present study. Future research should therefore incorporate instrumental postural control measures, such as stabilometric analysis, trunk control assessment, or motion analysis systems, to further investigate construct validity. Furthermore, the study sample included heterogeneous clinical groups, particularly within the neurological population. Variability in diagnoses, levels of motor impairment, and functional abilities may have influenced the observed results and contributed to within-group variability. Moreover, reliability was assessed under controlled observational conditions using trained evaluators familiar with the BMPI framework. Therefore, reliability estimates obtained in routine clinical settings may differ and should be investigated in future real-world implementation studies.

Additionally, the BMPI is based on ordinal item scoring, whereas several statistical analyses were performed using summed total scores treated as continuous variables. Although this approach is commonly applied in preliminary psychometric validation studies involving multidimensional clinical scales, it may introduce certain methodological limitations. Future studies should therefore consider the application of ordinal-specific analytical approaches, such as Rasch analysis or item response theory models, to further refine the measurement properties of the instrument.

In addition, although the BMPI was conceptually organized into four observational domains, the present study was not designed to perform advanced dimensionality analyses such as exploratory or confirmatory factor analysis. Similarly, internal consistency measures were not prioritized at this preliminary stage, as the BMPI was developed to assess complementary dimensions of respiratory motor organization rather than a strictly unidimensional construct. Future studies should further investigate the internal structure and dimensionality of the instrument using advanced psychometric approaches.

Finally, the cross-sectional design of the study limits the ability to assess developmental trajectories and responsiveness to intervention. Although the BMPI was conceptually developed with potential longitudinal applications in mind, longitudinal and intervention-based studies are required to determine whether the BMPI is sensitive to developmental and therapeutic changes and to evaluate its responsiveness following rehabilitation interventions. Overall, these considerations suggest that, while the BMPI demonstrates promising measurement properties, further research is needed to refine the instrument and to better understand its clinical and physiological relevance.

### 4.7. Study Limitations

Several limitations of the present study should be acknowledged.

First, the cross-sectional design does not allow for the assessment of developmental changes or the responsiveness of the BMPI to therapeutic interventions. Longitudinal and intervention-based studies are needed to determine whether the BMPI is sensitive to developmental and therapeutic changes over time and to evaluate its responsiveness following rehabilitation interventions.

Second, the assessment of construct validity was based on the relationship with GMFM-88, which, although widely used, does not directly capture respiratory or postural integration mechanisms. This may have influenced the strength of the observed associations.

Third, the absence of a gold standard measure for respiratory motor pattern organization limits the ability to perform criterion validation and to directly compare BMPI with existing instruments.

Fourth, the study sample included heterogeneous clinical populations, particularly within the neurological group, which may have contributed to variability in the results.

Although participants were recruited across predefined developmental age ranges, the present study was not designed to perform detailed age-stratified analyses of BMPI performance. Consequently, developmental trends in respiratory motor pattern organization across specific age groups were not explored in depth. Future studies should therefore investigate age-related differences and maturational trajectories of BMPI scores to better understand developmental changes in respiratory–postural integration during childhood.

Finally, the presence of floor and ceiling effects suggests potential limitations in the sensitivity of the scale at the extreme ends of performance.

These limitations should be considered when interpreting the findings and highlight the need for further research to confirm and extend the present results.

### 4.8. Clinical Implications

The findings of this study suggest that the BMPI may provide clinically relevant information regarding the organization of respiratory motor patterns in children.

As an observational tool that does not require specialized equipment, the BMPI may be particularly useful in routine clinical practice, where access to advanced diagnostic methods is often limited. The ability to assess breathing as part of a broader motor pattern may support a more integrative approach to pediatric physiotherapy, especially in populations with neurological or developmental disorders.

Importantly, the BMPI allows for the qualitative evaluation of breathing in relation to postural control and movement, rather than focusing solely on respiratory parameters. This may facilitate the identification of compensatory strategies, asymmetries, and context-dependent changes in breathing behaviors that are not captured by conventional respiratory assessments.

In clinical practice, the BMPI may be particularly useful as part of routine pediatric physiotherapy and neurorehabilitation assessment, especially in children presenting with developmental disorders, neurological conditions, or chronic respiratory dysfunction. Due to its observational and non-invasive nature, the tool may support longitudinal monitoring of respiratory motor organization over time and may facilitate individualized therapeutic planning by identifying compensatory breathing strategies, postural–respiratory interactions, and context-dependent changes in breathing behavior.

The results also suggest that the BMPI may be useful for monitoring changes over time, both in the context of natural development and in response to therapeutic interventions. This may be particularly relevant in pediatric populations, where functional changes are often gradual and multifactorial.

Taken together, the BMPI may contribute to expanding the scope of clinical assessment by incorporating respiratory motor organization as a relevant component of functional evaluation. However, further research is required to establish its role in clinical decision-making and treatment planning.

The BMPI may therefore represent a clinically accessible link between respiratory assessment and functional motor evaluation in pediatric practice. Nevertheless, although the instrument was developed around a theoretically defined four-domain structure, dimensionality analyses such as exploratory or confirmatory factor analysis were not performed in the present study. Therefore, further research is required to empirically verify the factorial structure of the instrument and confirm its dimensional organization.

## 5. Conclusions

The present study suggests that the Breath Motor Pattern Index (BMPI) may represent a reliable and clinically applicable tool for assessing respiratory motor pattern organization in children. The results indicate high inter-rater reliability and temporal stability, along with a relatively low measurement error, supporting the reproducibility of the assessment.

Construct validity analyses suggest that BMPI captures aspects of motor organization that are only partially reflected in global motor function measures and may differentiate between clinical populations with distinct functional characteristics. Taken together, these findings support the conceptualization of breathing as an integrated component of the motor system and highlight the potential value of incorporating respiratory motor pattern assessment into podiatric clinical evaluation. However, further research is required to confirm the sensitivity of the BMPI to developmental and therapeutic changes and to better define its role in clinical practice.

## Figures and Tables

**Table 1:** Characteristics of studies included in the scoping review.

Author (Year)	Study Design	Respiratory Assessment	Relevance to BMPI
Greer (2012) [10]	Narrative review	Central respiratory control	Fundamental basis for breathing as organized motor activity
Koos & Rajaee (2014) [6]	Narrative review	Fetal breathing activity	Supports early emergence of breathing as motor pattern
Juvin et al. (2022) [11]	Narrative review	Neural respiratory control	Strong neurophysiological integration evidence
Hodges et al. (2002) [12]	Experimental study	Diaphragm activity modulated by postural demands	Integration of breathing and postural control
PTJ study (2010) [4]	Experimental study	Respiratory timing	Task-dependent motor integration
Krupnik et al. (2015) [13]	Experimental study	Respiratory coordination	Functional integration in motor control
Hessler & Amazeen (2009) [14]	Experimental study	Respiratory timing	Breathing integrated with higher-level control
Barlow et al. (2006) [15]	Observational study	Pattern observation	Ontogenetic development of motor pattern
Hadders-Algra (2010) [4]	Narrative review	Not assessed	Supports adaptive nature of breathing pattern
Cavassini et al. (2022) [8]	Observational study	Thoracoabdominal kinematics	Strong clinical evidence of pattern disruption
Tabary & Rassler (2015) [16]	Experimental study	Breathing resistance manipulation	Strong evidence for interaction between respiratory and temporal organization of movement
Janssens L et al. (2024) [7]	Observational study	Breathing pattern assessment	Direct pediatric pattern evidence
Lauhkonen et al. (2019) [17]	Mini review	Structured light plethysmography	Supports observational pattern-based diagnostics
Ratnagiri et al. (2021) [18]	Observational/computational	Pattern classification	Strong evidence for objective pattern assessment
Rehouma et al. (2019) [19]	Pilot study	3D motion-based pattern analysis	Supports measurable coordination of breathing pattern

**Table 2:** Inter-rater reliability of the BMPI, including intraclass correlation coefficients (ICC), 95% confidence intervals (CI), and item-level agreement (weighted Cohen’s kappa).

**Pairwise Inter-Rater Reliability**			
**Domain**	**ICC (2.1) ^1^**	**95% CI ^2^**	**Interpretation**
BMPI total	0.998	0.996–0.999	Excellent
Domain B	0.991	0.983–0.996	Excellent
Domain T	0.993	0.985–0.999	Excellent
Domain P	0.999	0.997–1.000	Excellent
Domain C	0.997	0.993–0.999	Excellent
**Multi-Rater Subset (n = 10)**			
**Domain**		**ICC ^1^**	**95% CI ^2^**
BMPI total		0.999	0.995–1.000
Domain B		0.987	0.935–1.000
Domain T		0.997	0.981–1.000
Domain P		1.000	1.000–1.000
Domain C		1.000	1.000–1.000
**Item-Level Agreement (Weighted Cohen’s Kappa)**			
**Item**		**Weighted Kappa**	**Interpretation**
B1		0.961	Almost perfect
B2		0.981	Almost perfect
B3		0.975	Almost perfect
B4		0.965	Almost perfect
T1		0.984	Almost perfect
T2		0.983	Almost perfect
T3		0.961	Almost perfect
T4		0.992	Almost perfect
P1		1.000	Perfect
P2		1.000	Perfect
P3		1.000	Perfect
P4		0.983	Almost perfect
C1		0.992	Almost perfect
C2		0.983	Almost perfect
C3		0.974	Almost perfect
C4		1.000	Perfect

^1^ ICC: intraclass correlation coefficient (two-way random-effects model, absolute agreement, single measures); ^2^ CI: confidence interval. Weighted Cohen’s kappa was used for item-level agreement.

**Table 3:** Test–retest reliability of the BMPI, including intraclass correlation coefficients (ICC) and 95% confidence intervals (CI) for the total score and individual domains.

Measure	ICC ^1^	95% CI ^2^
BMPI total	0.999	0.998–1.000
Domain B	1.000	0.999–1.000
Domain T	0.999	0.997–1.000
Domain P	1.000	1.000–1.000
Domain C	1.000	1.000–1.000

^1^ intraclass correlation coefficients, ^2^ 95% confidence intervals.

## Data Availability

The data presented in this study are available on request from the corresponding author. The data are not publicly available due to ethical restrictions and the protection of sensitive clinical data of pediatric participants.

## Supplementary Materials

The following supporting information can be downloaded at: https://www.mdpi.com/article/10.3390/children13060759/s1. Table S1: Theoretical and neurophysiological framework of BMPI domains and corresponding clinical observations. Table S2: Theoretical and neurophysiological framework of BMPI domains and corresponding clinical observations. Table S3: Breath Motor Pattern Index (BMPI): observational domains and scoring framework.

## Abbreviations

The following abbreviations are used in this manuscript:

BMPI	Breath Motor Pattern Index
CI	Confidence Interval
COSMIN	Consensus-based Standards for the selection of health Measurement Instruments
GMFM-88	Gross Motor Function Measure-88
ICC	Intraclass Correlation Coefficient
IQR	Interquartile Range
MDC95	Minimal Detectable Change at the 95% Confidence Level
MeSH	Medical Subject Headings
PRISMA-ScR	Preferred Reporting Items for Systematic Reviews and Meta-Analyses Extension for Scoping Review
SD	Standard Deviation
SEM	Standard Error of Measurement
