# Supplementary material for: Development and Preliminary Validation of the Breath Motor Pattern Index (BMPI): An Observational Measure of Respiratory Pattern Quality in Children
Source: Children (Basel). 2026 May 29;13(6):759. doi: 10.3390/children13060759 (PMC13297550; doi:10.3390/children13060759)
Supplement: Supplementary file 1 [file children-13-00759-s001.zip › children-4306079-supplementary.pdf]

Supplementary Materials

For:

Development and preliminary validation of the Breath Motor Pattern Index (BMPI): an observational measure of respiratory pattern quality in children

Supplementary Table S1. Theoretical and neurophysiological framework of BMPI domains and corresponding clinical observations

BMPI domain	Theoretical / Neurophysiological Construct	Clinical Interpretation
Initiation and rhythmicity	Reflects the organization and continuity of respiratory neuromuscular activity generated by central respiratory control mechanisms. Includes temporal stability of the breathing cycle and smooth transition between inspiratory and expiratory phases.	Evaluates whether breathing is initiated smoothly and maintained with stable rhythm and temporal organization, without irregular pauses, abrupt tempo changes, or disrupted respiratory sequencing.
Thoraco-abdominal coordination	Represents multisegmental and multiplanar coordination of respiratory movement involving synchronized interaction between thoracic and abdominal compartments. Reflects biomechanical efficiency of respiratory pattern organization.	Assesses synchronization between thoracic and abdominal movement, symmetry of respiratory expansion, absence of paradoxical breathing, and balanced contribution of respiratory segments during breathing.
Adaptation to postural/task demands	Reflects context-dependent modulation of respiratory motor behavior in response to changing postural conditions and functional demands. Represents the adaptive integration of breathing within the motor system.	Evaluates the stability and adaptability of breathing during changes in body position and simple motor activities, including the ability to postural demands.
Integration with postural control and compensatory strategies	Reflects the interaction between respiratory function and postural stabilization mechanisms, including compensatory activation of accessory muscles and substitute motor strategies.	Assesses whether breathing remains integrated with postural control without excessive recruitment od accessory muscles, shoulder elevation, breath-holding, or compensatory stabilization strategies.

Supplementary Table S2. Theoretical and neurophysiological framework of BMPI domains and corresponding clinical observations

Database	Search string	Search date

PubMed	(“breathing” OR “respiratory pattern” OR “breathing pattern” OR “respiration”) AND (“motor development” OR “motor control” OR “motor pattern” OR “postural control” OR “posture”) AND (“child” OR “children” OR “pediatric” OR “pediatric” OR “infant”)	December 2025
Scopus	TITLE-ABS-KEY (“breathing” OR “respiratory pattern” OR “breathing pattern” OR “respiration”) AND TITLE-ABS-KEY (“motor development” OR “motor control” OR “motor pattern” OR “postural control” OR “posture”) AND TITLE-ABS-KEY (“child” OR “children” OR “pediatric” OR “pediatric” OR “infant”)	December 2025
Web of Science	TS=(“breathing” OR “respiratory pattern” OR “breathing pattern” OR “respiration”) AND TS=(“motor development” OR “motor control” OR “motor pattern” OR “postural control” OR “posture”) AND TS=(“child” OR “children” OR “pediatric” OR “pediatric” OR “infant”)	December 2025
Google Scholar	“breathing pattern” AND “postural control” AND child OR pediatric; “respiratory pattern” AND “motor development” AND children	December 2025

Additional screening procedures: Reference lists of included articles were additionally screened to identify potentially relevant publications not retrieved through database searching.

Eligibility criteria: Studies published between January 2000 and December 2025 involving pediatric populations (0–18 years) and addressing breathing in relation to motor development, postural control, or respiratory motor organization were considered eligible. Studies focusing exclusively on adult populations, isolated respiratory pathology without motor context, animal studies, or non-functional respiratory assessment were excluded.

Supplementary Table S3. Breath Motor Pattern Index (BMPI): observational domains and scoring framework.

**BMPI:** Breath Motor Pattern Index				
**BMPI domain**	**Observational item**	**0 points**	**1 point**	**2 points**
**Breathing initiation and rhythmicity**	Breathing initiation	abrupt, interrupted, or delayed initiation	partially smooth but inconsistent initiation	smooth and uninterrupted initiation
	Breathing rhythm stability	irregular rhythm with marked variability	partially stable rhythm with occasional irregularities	stable and regular breathing rhythm
	Inspiratory-expiratory transition	marked pauses or disrupted phase transition	mildly disrupted or inconsistent transition	smooth transition between phases
	Breathing tempo regulation	sudden unexplained tempo changes	occasional variability without clear functional adaptation	stable and context-appropriate tempo
**Thoraco-abdominal coordination**	Thoraco-abdominal synchrony	asynchronous or paradoxical movement	partially synchronized movement	synchronized thoracic and abdominal movement

	Segmental contribution	rigid dominance of one segment	partially balanced segmental contribution	harmonious contribution of thoracic and abdominal segments
	Paradoxical breathing	clearly present	intermittently present	absent
	Symmetry of movement	marked asymmetry	mild asymmetry	symmetrical respiratory movement
**Adaptation to postural and task demands**	Breathing stability during position changes	marked deterioration with position change	mild deterioration or inconsistency	stable breathing across positions
	Breathing during antigravity postures	substantial disruption	partially maintained organization	stable respiratory organization
	Breathing during simple motor activity	breathing disrupted by movement	partially integrated with movement	smoothly integrated with movement
	Context-dependent adaptability	no observable adaptation	inconsistent adaptation	effective adaptive modulation
**Integration with postural control and compensatory activity**	Accessory muscle activity	pronounced accessory muscle recruitment	moderate or intermittent recruitment	no excessive accessory muscle activity
	Shoulder elevation during inspiration	clearly present	intermittently present	absent
	Compensatory stabilization strategies	prominent compensatory strategies	occasional compensatory strategies	absence of compensatory stabilization
	Breath-holding during postural challenge	frequent breath-holding	occasional breath-holding	no visible breath-holding

Assessment instructions: The Breath Motor Pattern Index (BMPI) is an observational assessment tool based on the evaluation of spontaneous breathing under natural conditions without verbal breathing instructions. Assessment should be conducted in body positions associated with varying postural demands, including supine, sitting, and—when developmentally appropriate—standing or simple functional activity.
